# 

**Published:** 1876-07

**Authors:** 


*CONTENTS.*

| | Page |
|---|---|
| Cold Baths | 289 |
| A Simple Remedy | 290 |
| The Grahamite Theory | 291 |
| Save the Babies | 292 |
| Fat Folks | 294 |
| A Co-Operative Store in New York | 296 |
| See, Saw, Marjorie Daw (Poetry) | 297 |
| An Article by Dr. Hall | 299 |
| Spirometer | 301 |
| Paper for Water-Closets | 302 |
| Lost (Poetry) | 303 |
| Contracting Diseases | 305 |
| Depopulation | 307 |
| Poisonous Rooms | 308 |
| Constipation | 309 |
| Make Home Happy | 310 |
| Crinoline Dangers | 311 |
| Physical Culture | 312 |
| Fruit Season | 314 |
| Deaths in New York | 315 |
| From Laurie Todd's Note-Book | 315 |
| Position in Reading | 316 |
| Consumption Localities | 316 |
| Intussusception | 317 |
| A Model School-Room | 318 |
| Our Defences | 319 |
| Ways to Drunkenness | 319 |
| The Mystery at Silver Cliff | 321 |

